# Controlling the development of the dengue vector Aedes aegypti using HR3 RNAi transgenic *Chlamydomonas*

**DOI:** 10.1371/journal.pone.0240223

**Published:** 2020-10-14

**Authors:** Xiaowen Fei, Yang Zhang, Lili Ding, Yajun Li, Xiaodong Deng

**Affiliations:** 1 Department of Biochemistry and Molecular Biology, Hainan Medical College, Haikou, China; 2 Institute of Tropical Bioscience and Biotechnology, Chinese Academy of Tropical Agricultural Science, Haikou, China; 3 Hainan Provincial Key Laboratory for Functional Components Research and Utilization of Marine Bio-resources, Haikou, China; Faculty of Science, Ain Shams University (ASU), EGYPT

## Abstract

The *Aedes aegypti* mosquito plays an important role in the spread of diseases, including epidemic ones, such as dengue fever, Zika virus disease, yellow fever, and chikungunya disease. To control the population of *Ae*.*aegypti*, we transferred an HR3 RNAi fragment into the microalgae *Chlamydomonas*, which serves as food for *Ae*.*aegypti* larvae. Results showed that the HR3 RNAi transgenic algal strains were lethal to *Ae*.*aegypti*. The integumentary system of larvae fed with HR3 RNAi transgenic algal strains was severely damaged. Muscles of the larvae were unevenly distributed and disordered, and their midgut showed disintegration of the intestinal cavity. RNA-Seq results demonstrated that on the 4th day of inoculation with the transgenic algae, the abundance of early expressed genes in the 20E signal transduction pathway of larvae fed with the HR3 RNAi transgenic algal strain significantly reduced. These genes include E74, E75, E93, and 20E receptor complex EcR/USP and FTZ-F1 gene regulated by HR3. In later experiments, a scale test of 300 *Ae*.*aegypti* eggs per group was carried out for 30 days, and the survival rate of *Ae*.*aegypti* fed with the HR3 RNAi transgenic strain was only 1.3%. These results indicate that the HR3 RNAi transgenic strain exerts obvious insecticidal effect.

## Introduction

*Aedes* plays an important role in the transmission of mosquito-borne diseases. Among the world’s major epidemic mosquito-borne diseases, the most serious ones are dengue fever, Zika virus disease, yellow fever, and chikungunya disease, all of which are transmitted by *Aedes* mosquitoes. According to WHO statistics, about half of people worldwide are infected with these mosquito-borne diseases every year [1]. Since 2007, local transmission of dengue fever has been reported in 128 countries and regions in Asia, Africa, America, and Oceania. In China, the incidence of dengue fever is severe in China’s Guangdong, Yunnan, Guangxi, Fujian, Hainan, and Taiwan provinces. Statistical analysis of the Chinese Center for Disease Control and Prevention revealed 4662 cases of dengue fever in China in 2013, and the highest incidence was recorded in Guangdong with 2895 cases. Mosquito-borne diseases such as dengue fever have become a global public health issue [2–4].

Dengue fever is an acute arbovirus-borne disease caused by the dengue virus of the family *Flaviviridae* and genus *Flavivirus*. After mosquitoes bite a human body, the virus infects dendritic cells and macrophages near the biting site, proliferates through monocytes and capillary endothelial cells, and then invades the human circulatory and lymphatic system to form the first toxemia [5–6]. *Aedes* species, particularly *Ae*.*aegypti* and *A*. *albopictus*, are the main vectors of dengue virus, and the human body is its main source of infection [7]. Dengue fever is mainly caused by four serotypes of dengue viruses, all of which are pathogenic [8]. However, vaccines for these four serotypes have not yet been developed because of the heterogeneity and instability of the virulence of dengue virus. Nowadays, symptomatic treatment is the main method for managing dengue fever. Therefore, it is important to control the spread of dengue fever by cutting off the transmission route.

At present, chemical insecticides are still used to control *Ae*.*aegypti* and *A*. *albopictus*. However, this method not only causes irreversible pollution to the environment but also increases the tolerance of *Aedes* to chemical agents. Therefore, environmentally friendly technology that can control the two pathogenic *Aedes* species is urgently needed.

RNA interference (RNAi) technology refers to the silencing of target gene expression caused by the intervention of double-stranded RNA (dsRNA) [9,10]. This technology has a wide usage in mosquiotes control. Coy et al. silenced the V-ATPase A gene of *Aedes aegypti* mosquitoes through oral delivery of double-stranded RNA [11]. Zhang et al used Chitosan double-stranded RNA nanoparticle-mediated RNA interference to silence chitin synthase genes through larval feeding in the African malaria mosquito *Anopheles gambiae* [12]. Howevery, few reports by using RNAi transgenic algae to control the mosquito.

Mosquito larvae generally feed on tiny particles and microalgae that are smaller than their mouthparts, such as *Chlamydomonas*, *Chlorella*, and *Scenedesmus*. Based on preliminary feeding experiments, we found that the larvae of *Ae*.*aegypti* and *A*. *albopictus* can feed on *Chlamydomonas reinhardtii*, *Chlorella*, and *Scenedesmus*. Therefore, these algae may become vectors for double-stranded RNA transformation.

The hormone receptor 3 (HR3) gene plays an important role in insect metamorphosis. HR3 homologous genes have been identified in different species of insects, including *Drosophila*, *Ae*.*aegypti* [13], *Plutella tabaci*, *Ostrinia furnacalis*, tender moth caterpillar, *Chilo furnacalis*, *Tenebrio molitor*, *Helicoverpa armigera*, and *Blattella germanica* [14]. When ecdysone (20-hydroxyecdysone, 20E) is elevated in insects, it binds to ecdysone receptor EcR/USP, thereby activating regulatory factors BR, E74, and E75. Both E74 and E75 regulate the expression of the FTZ-F1 (fushi tarazu) gene mediated by regulating the expression of the HR3 gene and ultimately regulate the formation of molting and pupae [7]. Silencing of HR3 expression prevents the insects from undergoing normal molting and eventually causes larval death [8]. BR, E74, and E75 can also activate synthesis of yolk protein precursor (YPP) by binding to its promoter region, thereby promoting oocyte maturation. Studies on the HR3 gene knockout of *Ae*.*aegypti* showed that the synthesis of YPP is terminated and the oocyte of *Ae*.*aegypti* could not mature normally when the expression of HR3 is decreased, forming a dead embryo and resulting in the female *Ae*.*aegypti* failing to lay eggs [15]. Considering the important role of the HR3 gene in the reproduction and molting development of *Ae*.*aegypti*, we selected it as the target of RNAi silencing in the present study.

Currently, *Ae*.*aegypti* has been genome-wide sequenced [16], and the sequence of its HR3 [13] has also been reported. On this basis, in this study, the RNAi vector of HR3 was transformed into *Chlamydomonas*. Then, the confirmed engineered algal strain was fed to *Ae*.*aegypti* larvae. The growth and death of the larva and the observation of its anatomy and histomorphology were analyzed. Here is the report.

## Materials and methods

### Algal strains and culture conditions

The *C*. *reinhardtii* strain CC425 (cw15 arg2) was grown in TAP medium supplemented with 250 μg mL^-1^ arginine [17,18]. Liquid cultures were grown under continuous light of 150 μmol m^-2^ s^-1^ at 25°C under shaking conditions at 200 rpm. Strains on TAP-agar plates were incubated at a light intensity of 100 μmol m^-2^ s^-1^ at 22 C [19].

### Bioinformatics analysis

The HR3 protein domains were determined from the *Ae*.*aegypti* HR3 protein sequence downloaded from The *Aedes aegypti* genomics database (https://www.vectorbase.org/organisms/aedes-aegypti). The homology and otherlogy protein sequences of HR3 were analyzed for recognizable domains using the NCBI Batch conserved domain search tool (http://www.ncbi.nlm.nih.gov/Structure/bwrpsb/bwrpsb.cgi). Domains were also verified using the HMMER-based SMART website (http://smart.embl-heidelberg.de) and PFAM website (http://pfam.sanger.ac.uk/). A schematic diagram of the protein domain structures with functional domains was constructed using DOG 2.0 (Domain Graph, version 2.0) (http://dog.biocuckoo.org/) [20]. Protein alignment and phylogenetic analysis were aligned in the multiple sequence alignment tool CLUSTALX2.0 by using Gonnet Protein Weight Matrix with default parameters [21]. A phylogenetic tree was constructed by the Neighbor–Joining algorithm using MEGA 6.0. The bootstrap consensus tree was inferred from 1000 replicates.

### Construction of pMaa7 IR/HR3IR, an RNAi expression vector of the HR3 gene

The cDNA from the total RNA of *Ae*.*aegypti* was used as the template, and the HR3 RNAi fragment was amplified using HR3RNAIF (ATTTGCGCTAACATGCTATCG) and HR3RNAIR (CAGCCATTTCAAGTTCACTACG) as primers. The amplified fragment was inserted into pMD18-T to obtain pMD- HR3. The plasmid was sent to Shanghai Shenggong Biotechnology Co., Ltd. for sequencing. Then, the fragments were digested by HindIII/BamHIand XbaI/SalI and subsequently inserted into the corresponding cloning sites of plasmid T282 to acquire T282-HR3. Both RNAi vector of pMaa7 IR/XIR and intermediate vector of T282-HR3 were digested by EcoRI and then ligated to produce the RNAi recombinant pMaa7 IR/HR3IR [22].

### Algae transformation

The cells used for transformation (*C*. *reinhardtii* strain CC425 [cw15 arg2]) were grown to a density of 1–2 × 10^6^ cells mL^-1^, and the constructs were introduced into the cells by using the glass bead method [23]. The cells were collected through centrifugation and then washed twice before their resuspension in the TAP medium without arginine to a cell density of approximately 1 × 10^8^ cells mL^-1^. Exactly 2 μg of plasmid DNA and 400 μL of cells were mixed with 100 μL of 20% polyethylene glycol and 300 mg of sterile glass beads. After the mixture was vortexed for 15 s, the cells were washed from the glass beads and subsequently plated on TAP agar with 1.5 mM L-tryptophan, 5 μg paromomycin ml-1, and 5 μM 5-fluoroindole [24]. To test the positive tranformants algea with HR3, we use the primers GATTTAAATGCCAGAAGGAG and CGCGGTGAGCACCGGAACGG for PCR amplification.

### Breeding and biological detection of Ae.aegypti mosquitoes

Fresh water was added to a large Petri dish in an incubator at a temperature of 26°C and a humidity of 75%, and a small amount of food was added. After 8 h, the residual feed that was not dissolved in water was sucked out. The filter paper containing the mosquito eggs was placed in water, and the hatched larvae were observed after 2 h of incubation. The oil film on the surface of the incubating water was scraped off on time every day. When the larvae became pupae, they were sucked out into a paper cup filled with clean water and then transferred to a mosquito cage. When the mosquito pupae developed into adult mosquitoes, they were fed sugar water.

The experimental design includes experimental groups and multiple control groups. Each group contains 10 larvae hatched from eggs. The larvae fed with the HR3 RNAi transgenic algal strains were set as the experimental groups, whereas the larvae fed with non-transgenic *C*. *reinhardtii* CC425 and fodder were used as controls. Trails were repeated three times. The mortality of the larvae was recorded every day. The surviving larvae were fed continuously until they became pupae and developed into mosquitoes. The length and pupation time of the larvae were recorded. Some larvae were embedded in paraffin, sectioned, stained, and then examined by light microscopy.

In the large-scale feeding experiment, 300 larvae hatched from eggs were selected for each treatment group and control group. The duration of the experiment is 30 days. The larvae fed with the transgenic strains HR3-C9 was set as the experimental group, whereas the larvae fed with non-transgenic *C*. *reinhardtii* CC425 and fodder were used as controls. The survival rate of larvae and adults mosquito were recorded.

### Treatment of RNA-Seq samples of Larvae

*C*. *reinhardtii* CC425 and pMaa7IR/HR3IR transgenic strain HR3-C9 were inoculated into 50 mL of TAP liquid medium and shaken to the middle of the logarithmic growth phase. The same amount of algae was fed to *Ae*.*aegypti* larva every day. On the 4th and 6th days after feeding, the larvae were collected by centrifugation and frozen rapidly in liquid nitrogen. Total RNA of larvae was extracted, and the purity of the RNA was analyzed to meet the requirements of RNA-Seq sequencing. RNA-Seq was conducted by Illumina HiSeqTM 2500, and the results were plotted as heat maps by alignment statistics and gene differential expression analysis.

### HR3 gene expression detction of Aedae larvae

The endogenous gene expression of HR3 levels was detected by isolating total RNA from 3rd instar larva that were collected 10–20, and performing Real time PCR. Expression levels of HR3 were determined as compared to the endogenous control, *Ae*.*aegypti* RPS17(Ribo ribosomal protein S17, GenBank accession no. AAEL004175 (KY000705)). LarvaRNA was isolated using the TRIzol Reagent (Takara). Single-strand cDNA was synthesized from total RNA using oligo-dT primers in Bio-Rad Iscript-selected cDNA synthesis kit. Real-time PCR was performed on a BioRad iCycler iQ Real-Time PCR Detection System using SYBR Green as a fluorescent dye. Primers for RPS17 amplification are AAGAAGTGGCCATCATTCCA and GGTCTCCGGGTCGACTTC. The primer sequences for HR3 gene quantification are ATTTGCGCTAACATGCTATCG and CAGCCATTTCAAGTTCACTACG. The amplification rate of each transcript (Ct) was calculated by the PCR baseline subtracted method performed in the iCycler software at a constant fluorescence level. Cts was determined over three repeats. Relative fold differences were calculated using the relative quantification analytical method (2^-ΔΔCT^) with endogenous control [25].

### Statistical analyses

Data are presented as mean ± S.D. Duncan’s multiple range tests and T-test were performed to examine significant differences between means. In all cases, differences showing *P* < 0.05 were considered significant.

## Compliance with ethical standards

### Conflict of interest

The authors declare that there is no conflict of interests regarding the publication of this paper.

### Ethical approval

The laboratory research was conducted at the Chinese Academy of Tropical Agricultural Science. All methods met the ethical requirements of the institute, and followed guidelines of the Committee of Publication Ethics.

### Research involving human participants and/or animals

This article does not involve any studies with human participants or vertebrate animals.

## Results

### Bioinformatics analysis results

**Fig 1:**
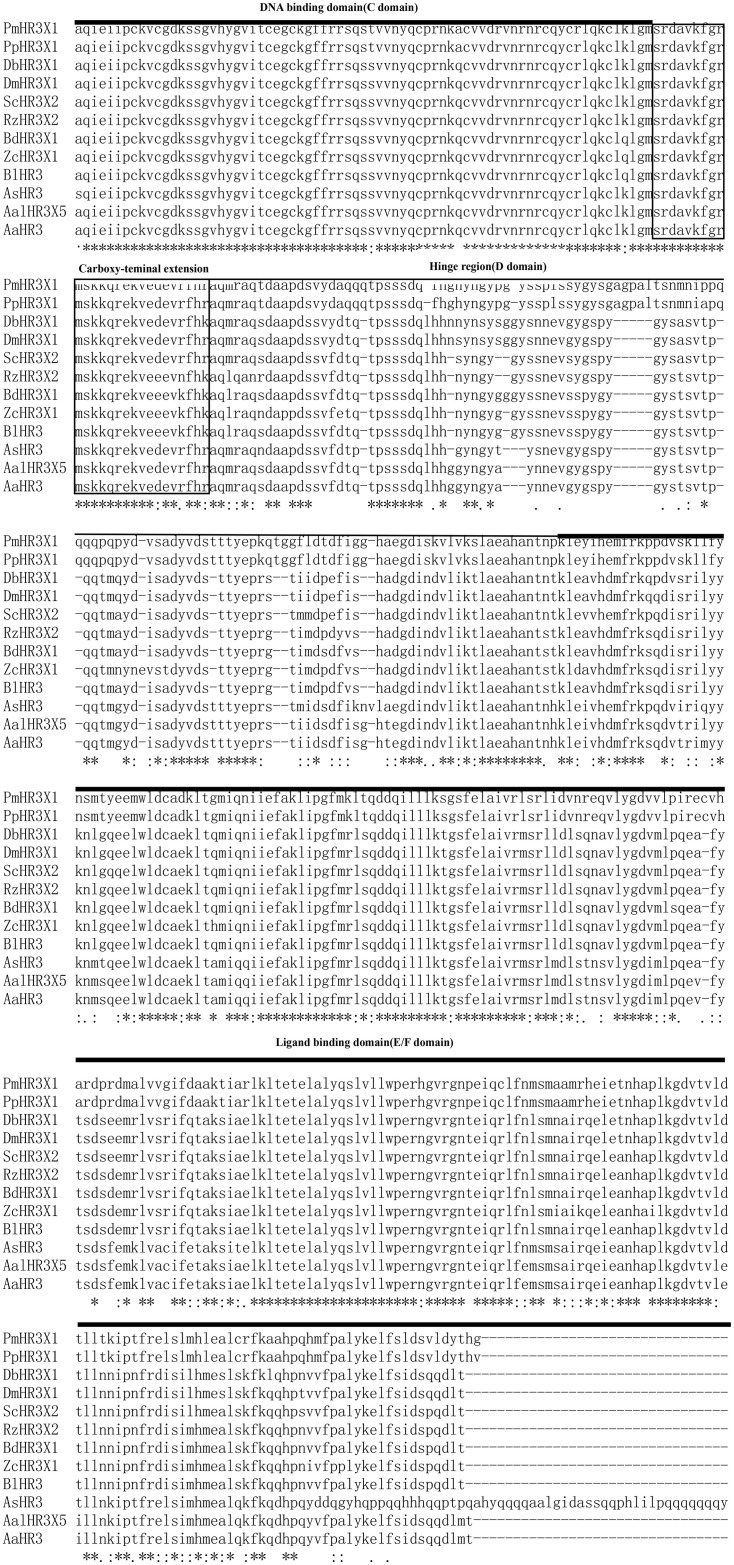
Amino acid sequence alignment of Aedes aegypti HR3 with its homologs. Sequences are from Papilio machaon (PmHR3X1, XP_014364014), Papilio polytes (PpHR3X1, XP_013136657), Drosophila biarmipes (DbHR3X1, XP_016947144), Drosophila Miranda (DmHR3X1, XP_017149048), Stomoxys calcitrans (ScHR3X2, XP_013103855), Rhagoletis zephyria (RZHR3X2, XP_017466500), Bactrocera dorsalis (BdHR3X1, XP_019847140), Zeugodacus cucurbitae (ZcHR3X1, XP_011182274), Bactrocera latifrons (BlHR3, XP_018799932), Anopheles sinensis (AsHR3, KFB44899), Aedes albopictus (AalHR3X5, XP_019549827), and Aedes aegypti (AaHR3, AF230281, AAEL009588-PA). The DNA-binding domain (C domain), hinge region (D domain), and ligand-binding domain (E/F domain) identified in D. melanogaster (Hu et al., 2003) are marked above the sequences.

**Fig 2:**
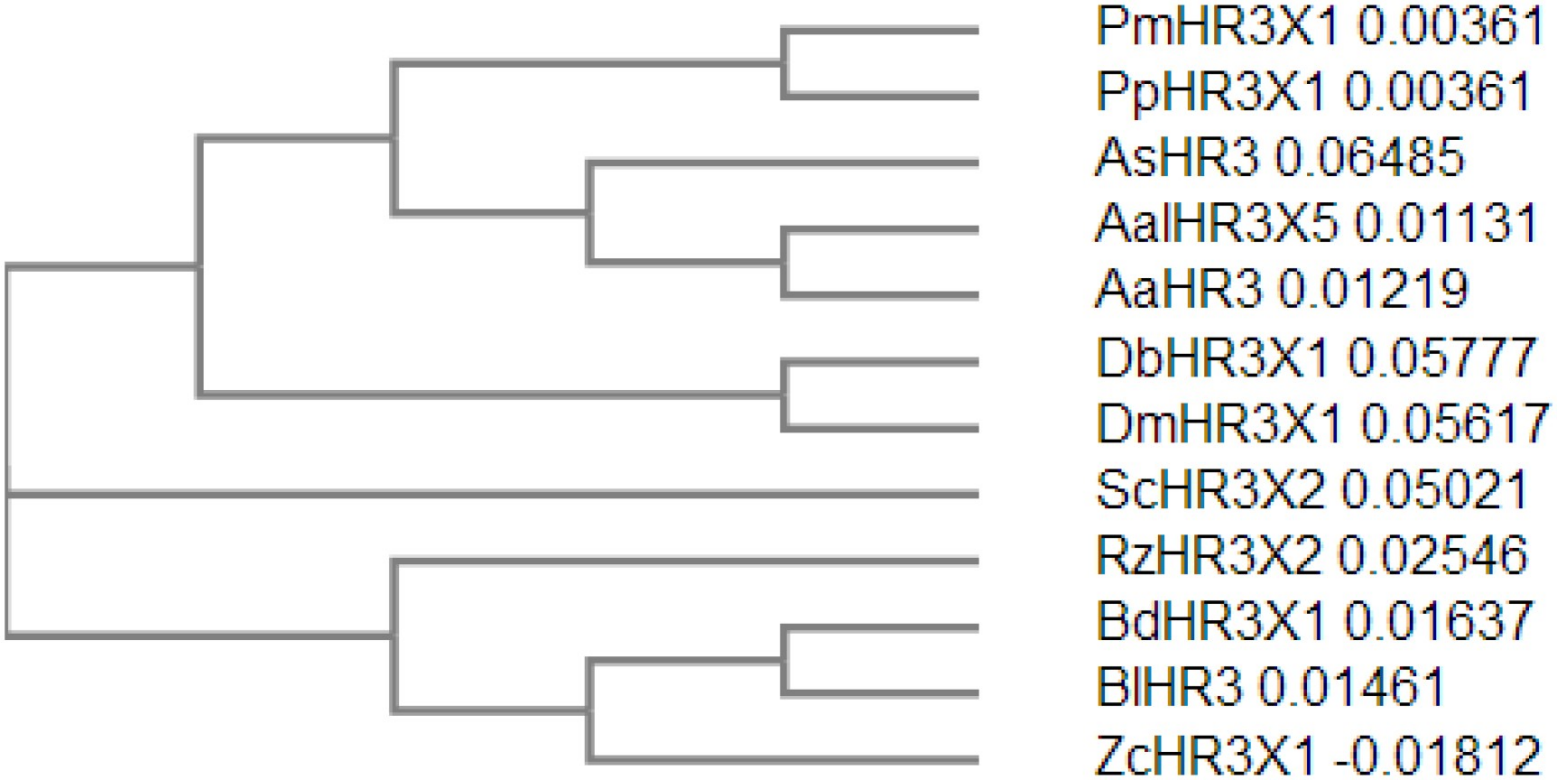
Phylogenetic tree of Aedes aegypti HR3 with its homologs. Protein sequences are from Papilio machaon (PmHR3X1, XP_014364014), Papilio polytes (PpHR3X1, XP_013136657), Drosophila biarmipes (DbHR3X1, XP_016947144), Drosophila miranda (DmHR3X1, XP_017149048), Stomoxys calcitrans (ScHR3X2, XP_013103855), Rhagoletis zephyria (RZHR3X2, XP_017466500), Bactrocera dorsalis (BdHR3X1, XP_019847140), Zeugodacus cucurbitae (ZcHR3X1, XP_011182274), Bactrocera latifrons (BlHR3, XP_018799932), Anopheles sinensis (AsHR3, KFB44899), Aedes albopictus (AalHR3X5, XP_019549827), and Aedes aegypti (AaHR3, AF230281, AAEL009588-PA). Neighbor-joining phylogenetic tree was constructed based on protein alignments of HR3 proteins using ClustalX2.

The open reading frame of the *Ae*.*aegypti* HR3 gene (GenBank accession number: AF230281, *Ae*.*aegypti* database: AAEL009588-PA) is 1407 bp in length, encoding 468 amino acid residues, and its 5′ non-coding region is 54 bp in length. It contains two C4-type zinc fingers with orphan receptors and hormone receptor functions. Compared with HR3 homologous genes in other insects, as shown in Fig 1, the HR3 protein of *Ae*.*aegypti* contains a DNA-binding domain at the N-terminal end and a ligand-binding domain at the C-terminal end. Mega6 cluster analysis showed that the HR3 protein sequence of *Ae*.*aegypti* has the highest homology (99%) with *A*. *albopictus (XP_019549827)*, followed by *Anopheles sinensis (KFB44899)*, *Papilio machaon (XP_014364014)*, *and Papilio polytes (XP_013136657)*. However, the homology was the lowest (71%) with *Zeugodacus cucurbitae (XP_011182274)* (Fig 2).

### pMaa7IR/HR3IR transgenic algal strains are lethal to *Ae*.*aegypti*

Reverse transcription of the total RNA from *Ae*.*aegypti* was used as a template to amplify the HR3 RNAi interference fragment, and a band of about 362 bp was obtained. This fragment was cloned into pMD18-T to obtain pMD-HR3. Sequencing results showed that the cloned HR3 fragment shared 100% homology with the *Ae*.*aegypti* HR3 gene. After the recombinant RNAi vector of pMaa7IR/HR3IR was transformed into *C*. *reinhardtii* CC425, 38 transgenic algal strains were identified as positive and used for further studies.

**Fig 3:**
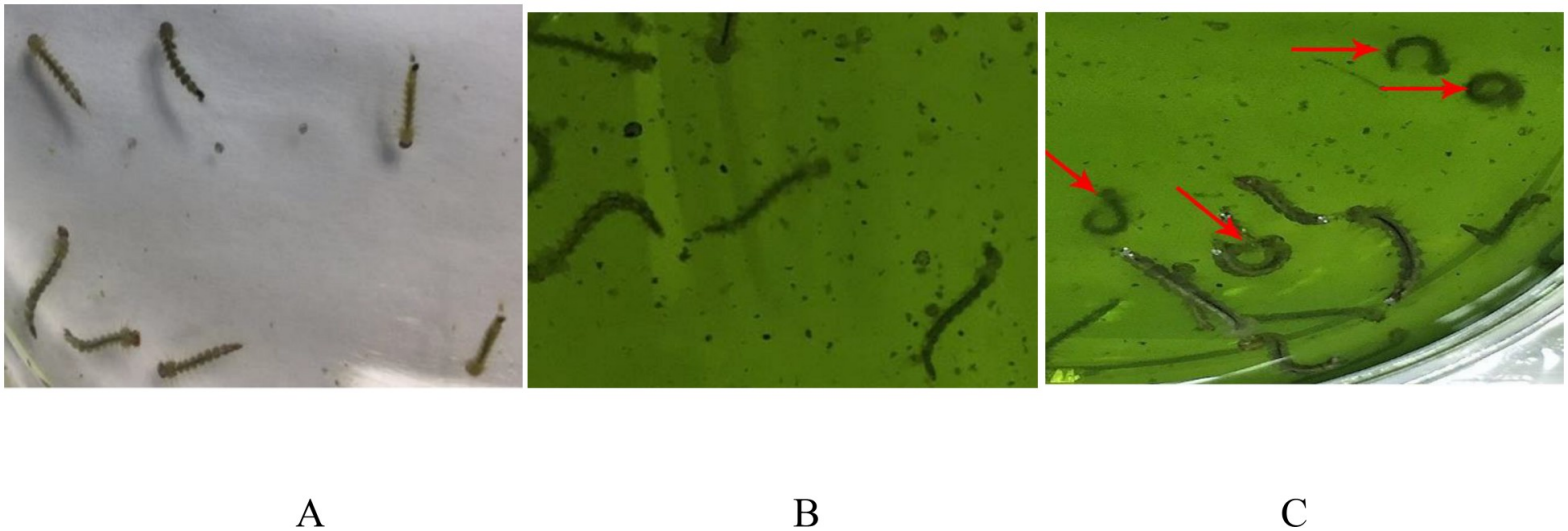
The growth status of Aedes aegypti larvae fed with pMaa7IR/HR3IR transgenic algae. A, Aedes larvae fed with fodder. B, Aedes larvae fed with non-transgenic C. reinharditii strain CC425. C, Aedes larva fed with pMaa7IR/HR3IR transgenic algae strain HR3-C9. The arrow points to the Aedes bit its anal fistula. The larvae bit their anal fistula in the early stage before death. Their bodies were floating on the water surface and trembling with active movements. In the late stage, their bodies were inactive and eventually sank into the water (C). The control group (larvae fed with non-transgenic C. reinhardtii CC425 and fodder) had stable growth with no biting and active movement (A, B).

When fed with the pMaa7IR/HR3IR transgenic algal strain, almost all the larvae bit their anal fistula in the early stage before death. Their bodies were floating on the water surface and trembling with active movements. In the late stage, their bodies were inactive and eventually sank into the water. The negative control group (larvae fed with non-transgenic *C*. *reinhardtii* CC425 and fodder) had stable growth with no biting and active movement (Fig 3).

**Fig 4:**
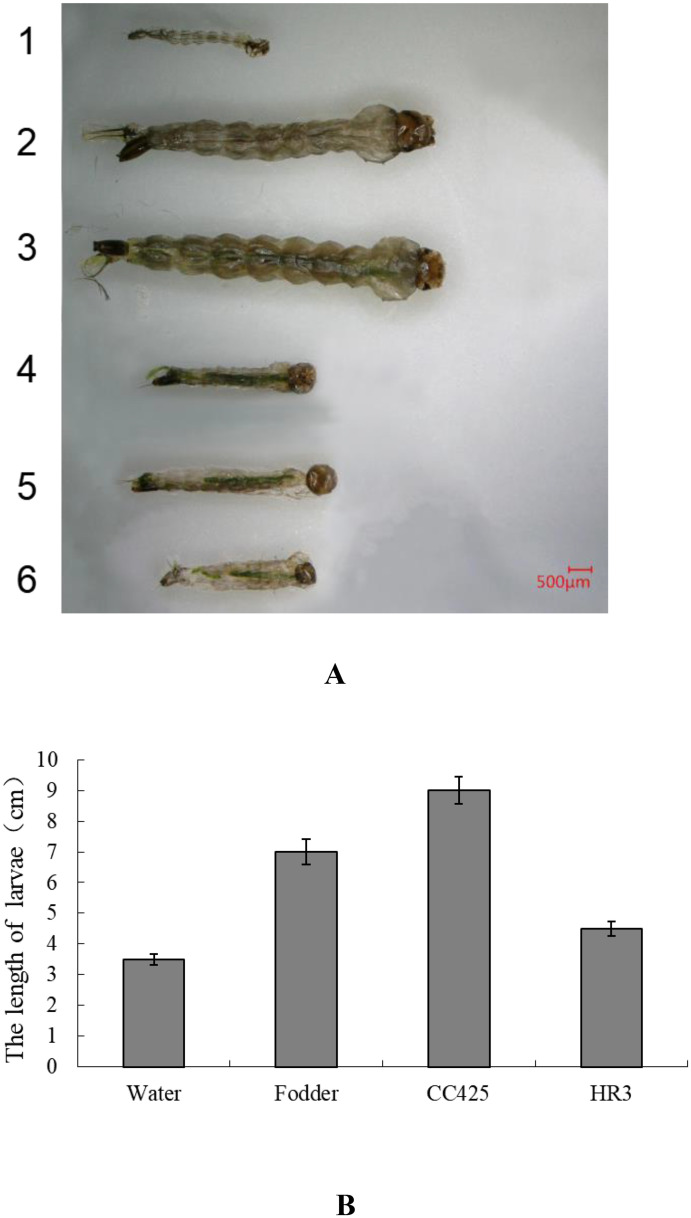
The length of Aedes aegypti larvae fed different foods. A: Observation of the size of larvae under an optical microscope. of them, 1, water: Aedes larvae fed with water; 2, fodder: Aedes larvae fed with fodder; 3,CC425: Aedes larvae fed with non-transgenic C. reinharditii strain CC425, 4, HR3: Aedes larva fed with pMaa7IR/HR3IR transgenic algae strain HR3-C9. B: the data of the body length of Aedes larvae fed with different food.

The length of the 4th-instar larvae were tested. The treated group fed with pMaa7IR/HR3IR transgenic strain HR3-C7 was 4259 ± 66 μm. However, the controls including group fed with fodder was 7876±78 μm, fed with non-transgenic *C*. *reinhardtii* CC425 was 8689 ± 81 μm (Fig 4). The larvae fed with pMaa7IR/HR3IR transgenic strain had a 51.1% reduction in body length compared with the larvae fed with *C*. *reinhardtii* CC425. These results indicate that feeding the larvae with the HR3 RNAi transgenic algal strain largely affected their growth.

**Fig 5:**
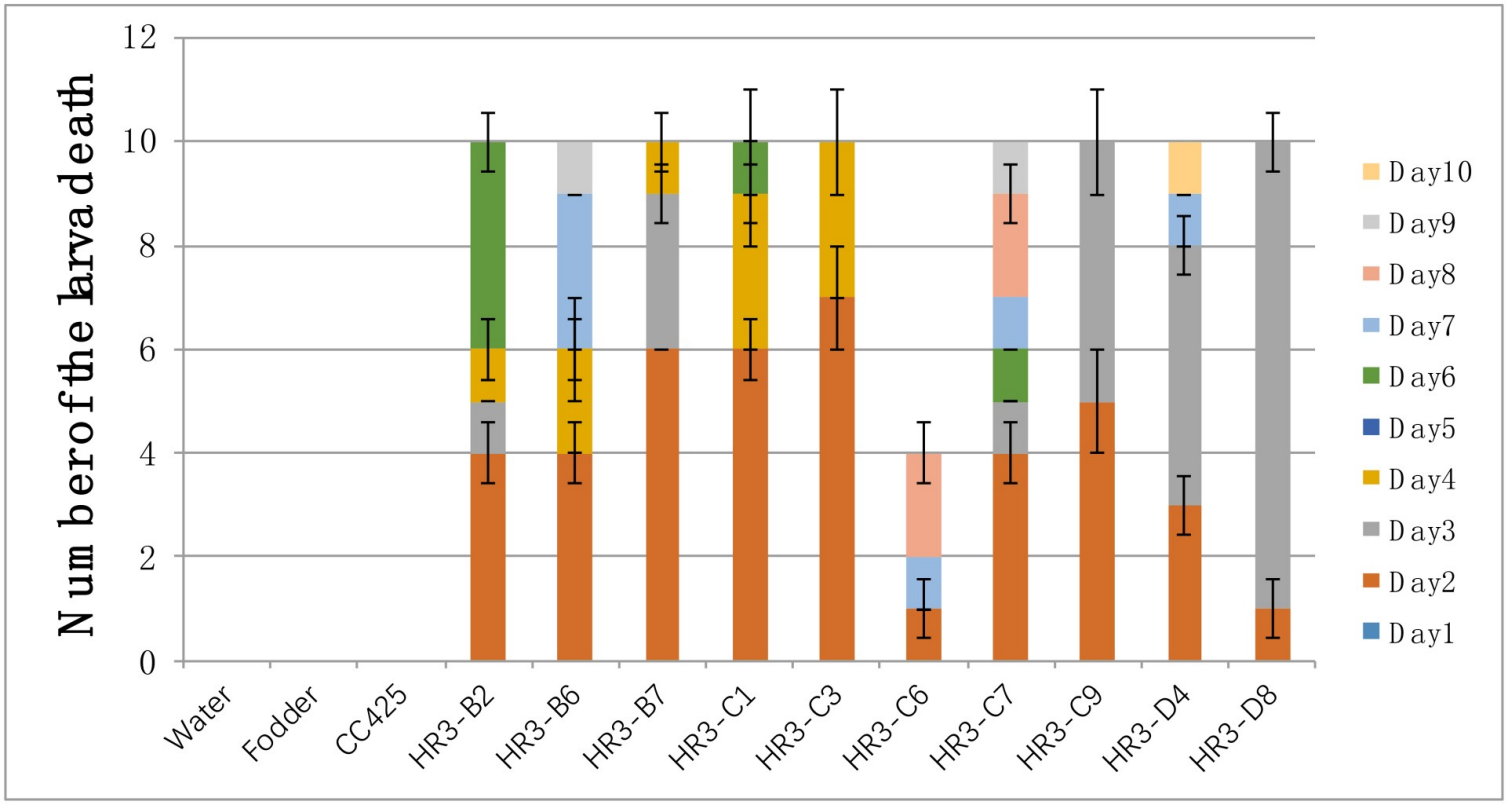
Statistics on the death of the Aedes aegypti larvae fed with HR3 transgenic algae. W: Aedes fed with water; F: Aedes fed with fodder; CC425: Aedes fed with non-transgenic C. reinharditii strain CC425; Maa7: Aedes fed with C. reinharditii CC425 transformed with Maa7IR/XIR; HR3-B2 to HR3-D8: Aedes fed with Maa7IR/HR3IR transgenic C. reinharditii strains. Note: 10 Aedes larvae per group, repeated 3 times, calculate the average to get the result.

Under the same feeding conditions, compared with control groups (fed with water, fodder, and *C*. *reinhardtii* CC425), the larvae fed with pMaa7IR/HR3IR transgenic algae died as early as the 2nd day. All tested larvae fed with transgenic algal strain HR3-C9 or HR3-D8 died on the 3rd day. Except for HR3-C6, all the other larvae fed with pMaa7IR/HR3IR transgenic algae died on the 10th day at the latest. This result indicates that the pMaa7IR/HR3IR genetically engineered algae exert a serious lethal effect on *Ae*.*aegypti* (Fig 5).

### HR3 mRNA levels in *Ae*.*aegypti* larvae fed with HR3 RNAi transgenic alage were significantly reduced

**Fig 6:**
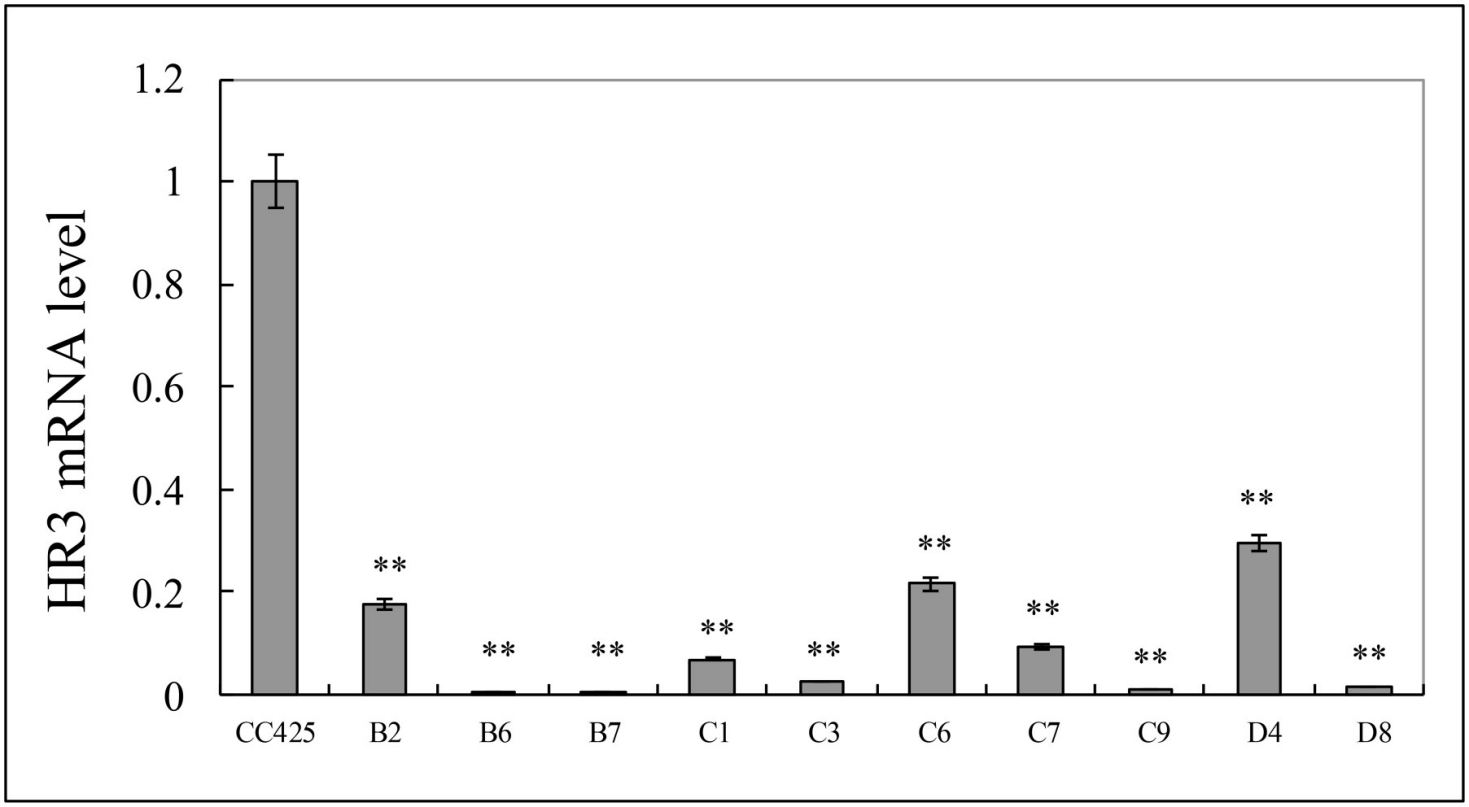
The relative HR3 mRNA level of Aedes aegypti larvae after fed with the pMaa7IR/HR3IR transgenic algae after 6 days. CC425: Aedes fed with non-transgenic C. reinharditii strain CC425; B2 to D8: Aedes fed with pMaa7IR/HR3IR transgenic C. reinharditii strains. Note: 10 Aedes larvae per group, repeated 3 times, calculate the average to get the result.

To detect the expression of the HR3 gene in *Ae*.*aegypti* larvae fed with transgenic engineered algae, we tested the cells by real-time PCR on the 6th day of larval incubation using *Ae*.*aegypti* fed with *C*. *reinhardtii* CC425 as a control. Compared with that of the control, the HR3 gene expression level of the *Ae*.*aegypti* larvae fed with the pMaa7IR/HR3IR transgenic engineered strain decreased significantly (Fig 6). The expression of HR3 in the larvae fed with HR3-B7 decreased by 99.7% compared with the control. The HR3 expression of the larvae fed with HR3-B6 and HR3-C9 decreased by 99.5% and 99.2%, respectively. The HR3 expression of the other larvae fed with HR3-B3, HR3-C3, and HR3-C7 decreased by 82.6%, 97.6%, and 91.1%, respectively. The above results show that the engineered algal strains can effectively silence the HR3 gene in *Ae*.*aegypti*.

### Epidermis, muscle, and midgut tissues of larvae fed with HR3 RNAi transgenic algae were significantly damaged

To understand whether the tissues of *Ae*.*aegypti* larvae fed with HR3 RNAi transgenic algae show pathological changes that might cause subsequent death, we prepared paraffin sections of the larvae and performed microscopic observation. The anatomical structure of *Ae*.*aegypti* larvae consists of an epidermis of the head and body wall, a digestive system, a reproductive system, and a respiratory system.

**Fig 7:**
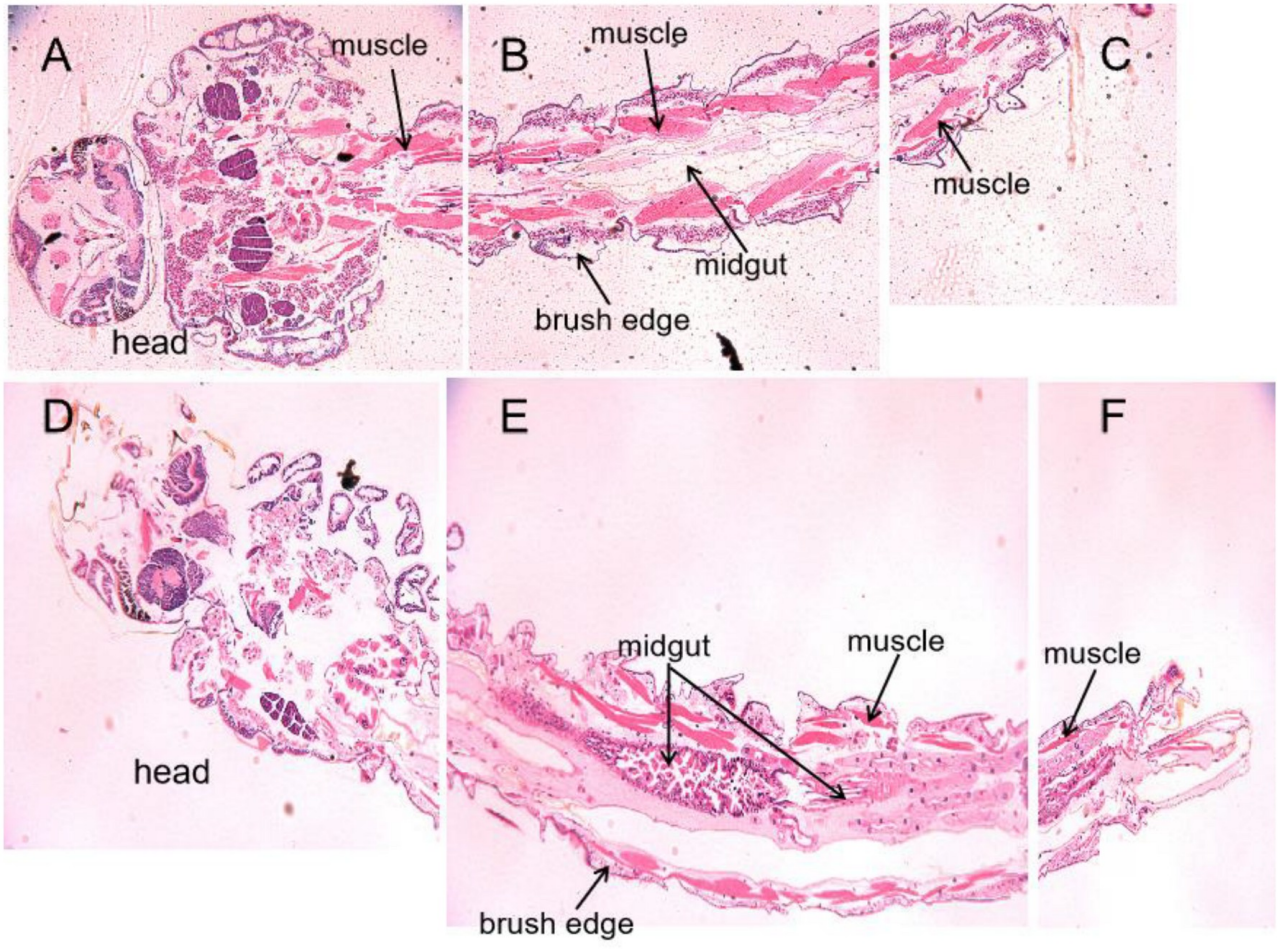
Tissue slices results of Aedes aegypti larvae fed with HR3 RNAi transgenic algae strain HR3-C9. A, B, C. The whole body of the larva fed with none-transgenic C. reinharditii strain CC425; D, E, F: The whole body of the larva fed with pMaa7IR/HR3IR transgenic algae strain HR3-C9. Larvae fed with C. reinhardtii CC425 had intact epidermis, thick muscles, and clear lines. Their midgut was normal and intestinal lumen was intact (A, B, C). The integumentary system of the larvae fed with HR3 RNAi transgenic algae was severely damaged and Their brush edge disappeared. Muscles of them were unevenly distributed, disordered, and some had even dissolved. The midgut were disintegrated with vacuolation of some cells (E, F).

Tissue sections were observed under a stereomicroscope. Results showed that the larvae fed with non-transgenic *C*. *reinhardtii* CC425 had intact epidermis, thick muscles, uniform muscle distribution, and clear lines. Their midgut was normal and intestinal lumen was intact (Fig 7A–7C). In contrast, The integumentary system of the larvae fed with HR3 RNAi transgenic algal strains was severely damaged and Their brush edge disappeared. Muscles of them were unevenly distributed, disordered, and some had even dissolved. The abnormal morphology of their midgut were characterized by disintegration of the intestinal cavity and vacuolation of some cells. Moreover, the striate edge was detached (Fig 7E and 7F).

### RNA-Seq detection of metabolic pathway changes in Ae.aegypti larvae fed with HR3 RNAi transgenic algae

The larval RNA on the 4th and 6th days after fed with the transgenic algal strain was extracted and subjected to RNA-Seq. On day 4, the larvae fed with the pMaa7IR/HR3IR strain expressed 988 upregulated genes, 2282 downregulated genes, and 9696 genes with no significant difference. On day 6, 775 genes were upregulated, 709 genes were downregulated, and 11,831 genes showed no significant difference. The expression of some genes involved in the metabolism of xenobiotics by the cytochrome P450 pathway, oxidative phosphorylation pathway, DNA replication, and RNA degradation pathway increased significantly. These genes include ① GST, GSTK1, HPGDS, and UGT in the metabolism of xenobiotics by the cytochrome P450 pathway; ② PCNC of DNA polymerase B, ε1 subunit of DNA polymerase ε and Mcm2, 3, 4, 5, 7, RFA2, 4, and RPA3 of the MCM complex in DNA replication; and ③ Ndufa1, Ndufb4, Ndufb11, Ndufb12, and Ndufs4 of DADH dehydrogenase and COX5A, COX6A, COX6B, COX7C, and COX11 of cytochrome C oxidase in the oxidative phosphorylation pathway. This result may be attributed to the toxic effect of the transgenic algal strain, resulting in a significant increase in gene expression in toxic degradation, DNA replication, and energy synthesis after the larvae were fed with the engineered algae.

**Fig 8:**
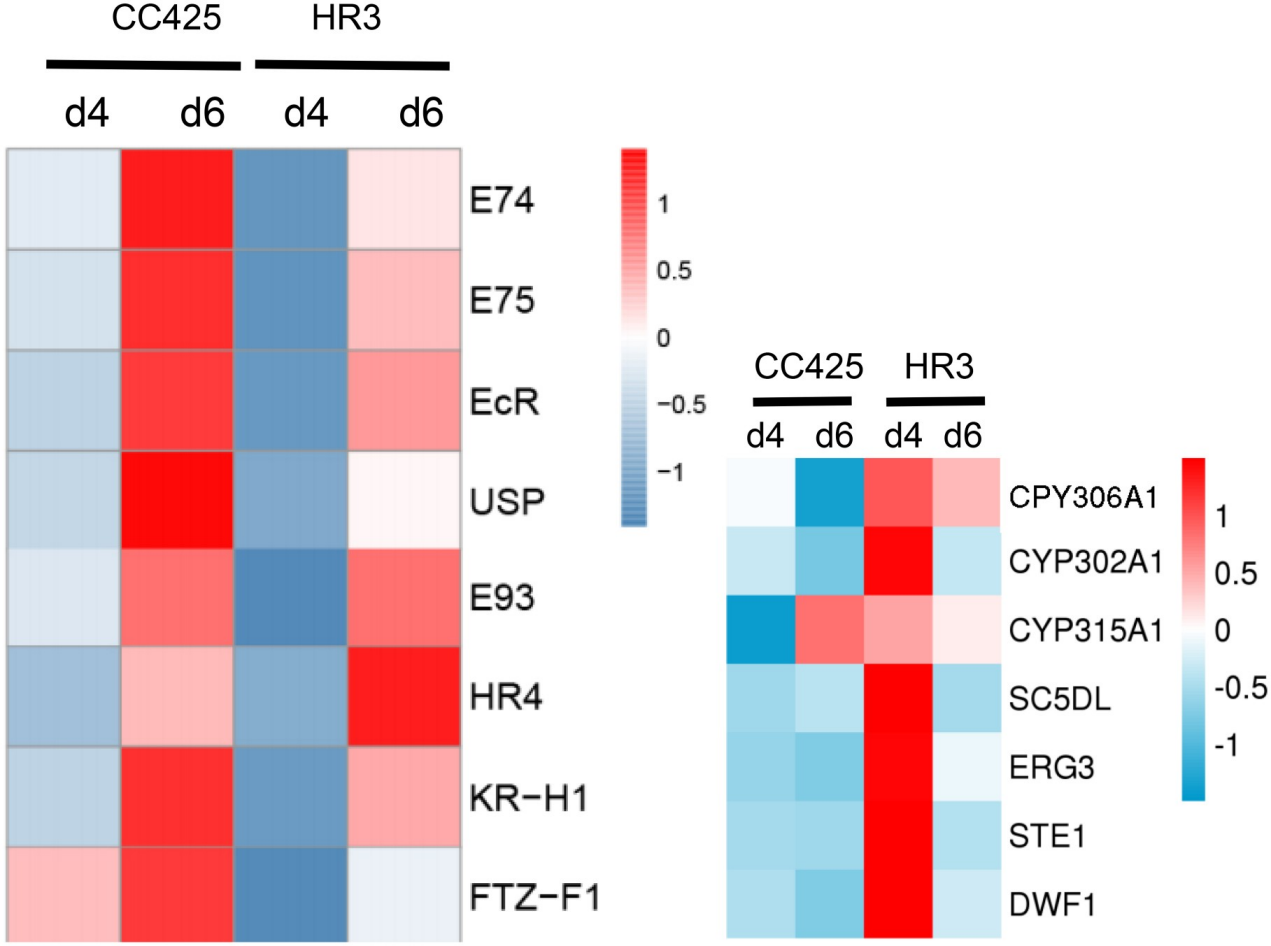
mRNA abundance of 20E signal transduction pathway, 20E synthesis, and cholesterol synthesis-related genes on the 4th and 6th day of larvae fed with pMaa7IR/HR3IR transgenic engineered strain. E74, LOC5579075; E75, LOC5569135; EcR, LOC5572184; USP, LOC5576337; E93, LOC5565069; HR4, LOC5579849; KR-H1, LOC5574499; FTZ-F1, LOC5573425. SC5DL, LOC110676479; ERG3, LOC 110681460; STE1, LOC 110676480; DWF1, LOC 5575872; CPY306A1, LOC110674175; CYP302A1, LOC5574850; CYP315A1, LOC5575470; d4, 4 days of Aedes fed with none-transgenic C. reinharditii strain CC425 or pMaa7IR/HR3IR transgenic algae strain HR3-C9; d6, 6 days of Aedes fed with none-transgenic C. reinharditii strain CC425 or pMaa7IR/HR3IR transgenic algae strain HR3-C9.

After the HR3 gene in the larvae was silenced by feeding with the pMaa7IR/HR3IR transgenic engineered algal strain (Fig 6), HR3 lost its regulation of downstream genes, which affected the metamorphic growth and formation of larvae, thus causing their death. The results of RNA-Seq showed that on the 4th day of incubation, the abundance of early expressed genes in the 20E signal transduction pathway of larvae fed with the pMaa7IR/HR3IR transgenic algal strain significantly reduced, including E74, E75, E93, and 20E receptor complex EcR/USP and FTZ-F1 gene regulated by HR3. In addition, the expression of late-stage genes, such as HR4 and KR-H1, decreased. On the 6th day of incubation, the mRNA abundance of E74, E75, EcR/USP, HR4, FTZ-F1, and KR-H1 decreased (Fig 8). We speculate that these genes are positively correlated with HR3 silencing.

Silencing of the HR3 gene in *Ae*.*aegypti* larvae also affects the anabolism of ecdysone. RNA-Seq results showed that the expression levels of genes associated with ecdysone E20 synthesis, such as CYP306A1, CYP302A1, and CYP315A1, increased significantly on the 4^th^ day of incubation. However, the downstream gene HR3 in the 20E signal transduction pathway was blocked. Thus, despite the high accumulation of 20E in vivo, it could not regulate the downstream functional gene expression by normal signal transduction, which resulted in delayed development and normal pupation. The normal pupation time of *Ae*.*aegypti* larvae is 6–8 days. Feeding with the pMaa7IR/HR3IR transgenic engineered algae can delay the larvae pupation by period up to 30 days, resulting in the majority not being pupated.

Given that silencing HR3 blocks the delivery of 20E, the synthesis of the 20E precursor, cholesterol, and its derivatives significantly increased in the steroid biosynthesis pathway. Fig 8 shows that the expression of key genes for the synthesis of cholesterol and its derivatives in the steroid biosynthesis pathway, such as SC5DL, ERG3, STE1, and DWF1, increased significantly on day 4.

### The HR3 RNAi transgenic algae exerts obvious insecticidal effect

**Fig 9:**
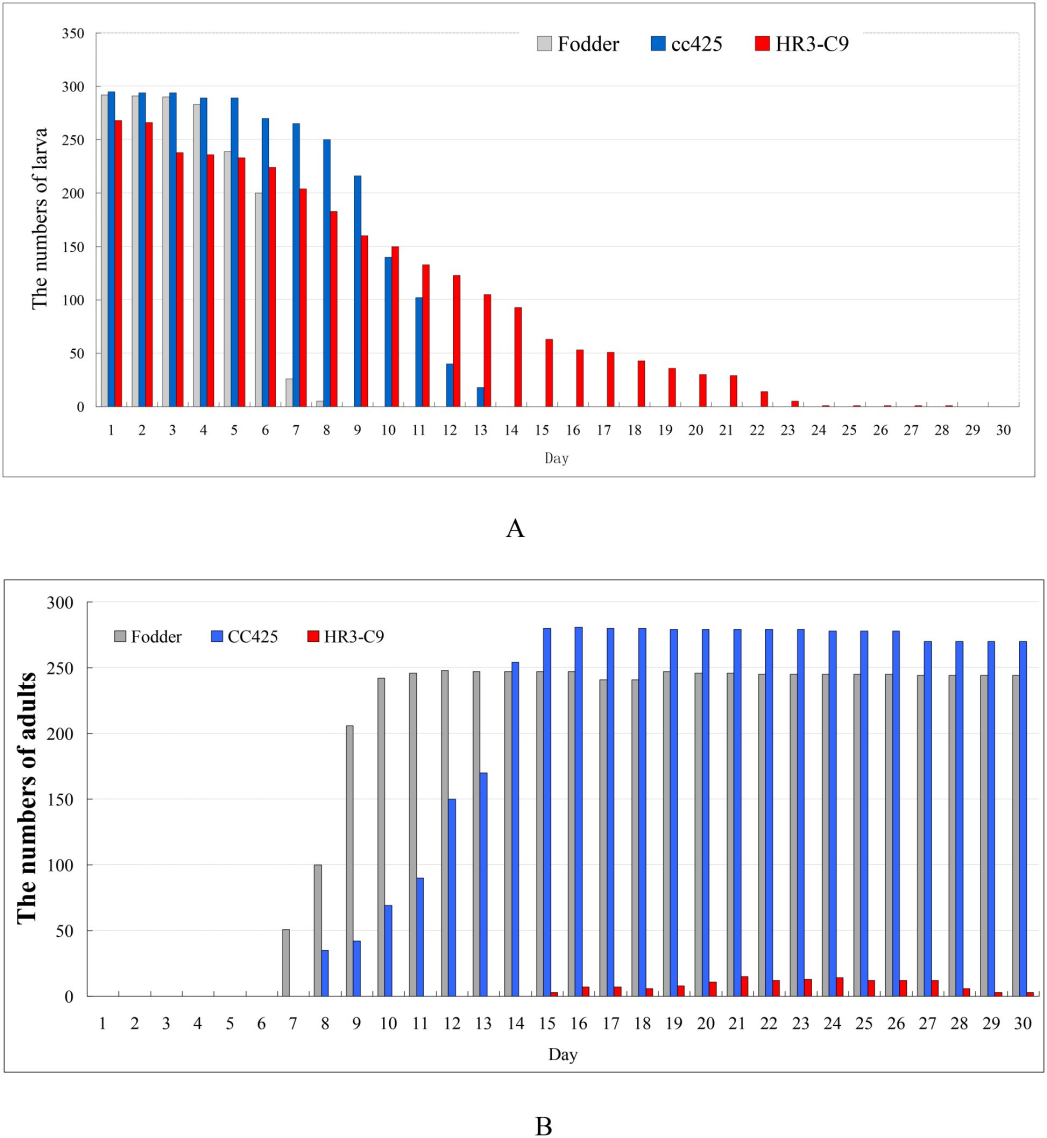
Survival rate of Aedes aegypti larvae (A) and adults (B) fed with the Maa7IR/HR3IR transgenic algae for 30 days. Fodder, Aedes fed with fodder; CC425, Aedes fed with none-transgenic Chlamydomonas reinhardtii strain CC425; HR3-C9, Aedes fed with pMaa7IR/HR3IR transgenic C. reinhardtii algal strain HR3-C9.

A scale test of 300 *Ae*.*aegypti* eggs per group was carried out for 30 days, and the data of biological observations were statistically analyzed. The data of larval hatching are as follows. In the control group fed with fodder, 292 eggs hatched. The larvae began to pupate on the 4th day, and all larvae completely pupated on the 8th day. In another control group fed with non-GM algae CC425, 295 eggs hatched. The larvae began to pupate on the 4th day, and all larvae pupated completely on the 13th day. In the experimental group fed with the transgenic pMaa7IR/HR3IR strain, 268 eggs hatched. The larvae began to pupate on the 4th day, and all larvae pupated completely on the 28th day (Fig 9A).

The survival rate of adult mosquitoes was as follows. The larvae fed with fodder completely developed into adults on the 12th day, and 244 adult mosquitoes survived on the 30th day, with a rate of 81.3%. On the 16th day, the larvae fed with CC425, a non-GM algal strain, completely developed into adults. On the 30th day, 270 mosquitoes survived, with a rate of 90%. The larvae fed with the transgenic pMaa7IR/HR3IR strain were completely developed into adults on the 24th day, and four mosquitoes survived on the 30th day. The survival rate was 1.3%, accompanied by uninterrupted death of larvae and adults. These results suggest that the transgenic pMaa7IR/HR3IR strain exerts obvious insecticidal effect (Fig 9B).

## Discussion

The use of biotechnology to control mosquitoes instead of traditional chemical pesticides is the future direction of vector mosquito control. At present, the use of Wolbachia infection and gene splicing male infertility techniques are relatively successful cases [26–33]. However, their disadvantage is that wild female mosquitoes prefer to mate with wild male mosquitoes in comparison to a Wolbachia bacterial infection male Aedes or a male Aedes that has been transferred with a lethal gene. The transgenic male sterile Aedes, code-named OX513A, which transgenic offspring have a survival rate of 15%–18% in outdoor after eating animal food, dog food, and cat food containing tetracycline residues [34–36]. This phenomenon undoubtedly poses a potential safety hazard for genetically modified organisms. Moreover, the obstacle to using *Wolbachia*’s mosquito-control technology is that *Wolbachia* has mutual transmission (horizontal transmission) in other insects, although reports of *Wolbachia* bacteria infecting mammals and humans are lacking [37–39]. *Wolbachia* not only spreads among the individuals of *Trichogramma* but also enables cross-species transmission between parasitic wasps and *Drosophila*. Whether *Wolbachia* can be transmitted to other organisms in the environment through mosquitoes, including predators of mosquitoes, is not conclusive.

Microalgae are a natural food of *Ae*.*aegypti* larvae and widely found in nature. At present, microalgae such as *Chlorella*, *Spirulina*, and *Chlamydomonas* can be industrialized and produced on a large scale. Compared with transgenic *Aedes* and *Aedes* infected by *Wolbachia*, the cost of production of transgenic microalgae is low and the technology is well-developed. It can be placed in a closed area for a long time to form dominant algae species, thereby gradually eliminating *Aedes* in corresponding areas. This method provides a new concept and method for using bio-insecticides to block the prevalence of malignant infectious diseases, such as dengue fever, Zika virus disease, and yellow fever.

**Fig 10:**
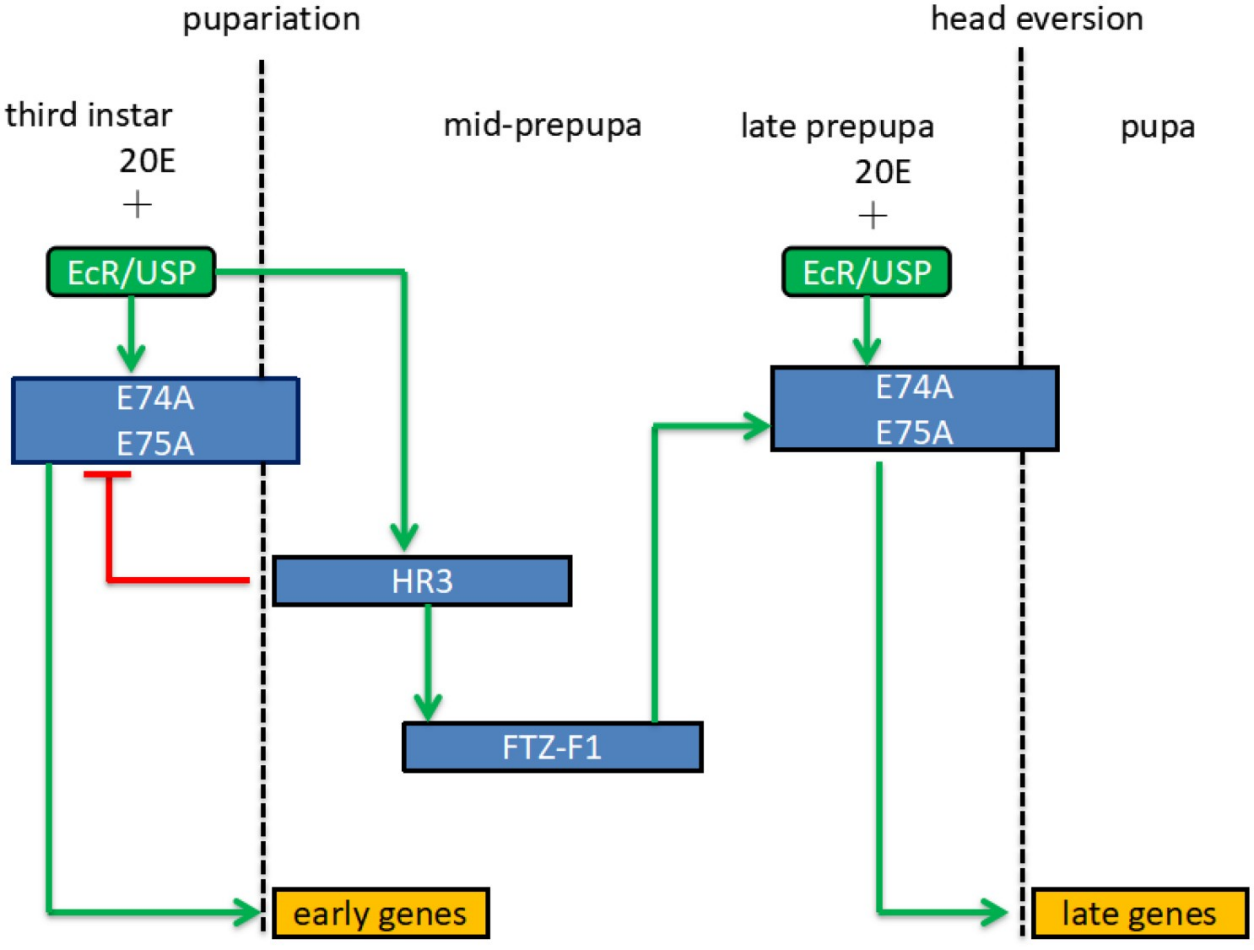
Schematic diagram of ecdysone 20E signal transduction. From the 3rd instar larva to the early pupation stage, 20E regulates the expression of E74A and E75A by binding to the receptor EcR/USP, thus initiating the early pupation-related gene expression. During the mid-prepupa period, 20E regulates HR3 expression by binding to the receptor EcR/USP, and HR3 accumulation inhibits the expression of E74A and E75A, thus turning off early pupation-related gene expression. During the late prepupa period, HR3 regulates the expression of E74A and E75A through FTZ-F1, thereby initiating the expression of late pupation-related genes.

In this study, we transferred an HR3 RNAi fragment into the microalgae Chlamydomonas, which serves as food for Ae.aegypti larvae. Results showed that the HR3 RNAi transgenic algae was lethal to Ae.aegypti larvae. The epidermis of larvae was severely damaged. Their muscles of the larvae were unevenly distributed and disordered, and their midgut showed disintegrated. HR3 is located in the ecdysone 20E signal transduction pathway. By regulating FTZ-F1, it can regulate E74 and E75, thereby regulating the pupation of Aedes mosquitoes (Fig 10). If the expression of HR3 is blocked, it will affect the expression of FTZ-F1, E74, E75 genes, which will affect the pupation of larvae and cause the death of larvae. The RNA-Seq test results showed that the expression of E74, E75, FTZ-F1 genes was greatly reduced due to the silence of HR3. The results of a large-scale experiment of 300 larvae in this study showed that the survival rate of Ae.aegypti fed with the HR3 RNAi transgenic strain was only 1.3%. These results indicate that the HR3 RNAi transgenic strain exerts obvious insecticidal effect. This result indicates that HR3 RNAi transgenic Algae strains have commercial application prospects. In the future, we will conduct in-depth research on the survival of algae strains in environmental waters, the impact on ecology and other organisms, and genetic safety.

## Appendix 1

### Amino acid sequences of *Aedes aegypti* HR3

1 mlrdapnrse lemavsstvf dsmlaqieii pckvcgdkss gvhygvitce gckgffrrsq

61 ssvvnyqcpr nkqcvvdrvn rnrcqycrlq kclklgmsrd avkfgrmskk qrekvedevr

121 fhraqmraqs daapdssvfd tqtpsssdql hhggyngyay nnevgygspy gystsvtpqq

181 tmgydisady vdstttyepr stiidsdfis ghtegdindv liktlaeaha ntnhkleivh

241 dmfrksqdvt rimyyknmsq eelwldcaek ltamiqqiie faklipgfmr lsqddqilll

301 ktgsfelaiv rmsrlmdlst nsvlygdiml pqevfytsds femklvacif etaksiaelk

361 ltetelalyq slvllwpern gvrgnteiqr lfemsmsair qeieanhapl kgdvtvleil

421 lnkiptfrel simhmealqk fkqdhpqyvf palykelfsi dsqqdlmt
